# Supplementary material for: Comparison of de-duplication methods used by WHO Global Antimicrobial Resistance Surveillance System (GLASS) and Japan Nosocomial Infections Surveillance (JANIS) in the surveillance of antimicrobial resistance
Source: PLoS One. 2020 Jun 26;15(6):e0228234. doi: 10.1371/journal.pone.0228234 (PMC7319286; doi:10.1371/journal.pone.0228234)
Supplement: S1 Fig — It stores and can analyze the data tabulated using the de-duplication in GLASS. Users can interact with these data by simply clicking their mouse, and create antibiograms stratified by the bed size of hospitals (under 200, over 200 and under 500, over 500 beds), inpatient or outpatient, gender, specimen type, and age group as defined by GLASS. Based on this, users can create antibiograms after excluding UNKNOWN_NO_AST. As another variable regarding missing data, GLASS defines UNKNOWN_NO_BREAKPOINTS representing the number of isolates where AST was performed but no interpretation of the results is available for a specific antibiotic. In the Excel tool, we have renamed this as UNKNOWN_NO_SIR to imply that S-I-R interpretation was not possible. It can be included or excluded from the denominator for calculating resistance rates in the tool. (DOCX) [file pone.0228234.s001.docx]

# Supporting information


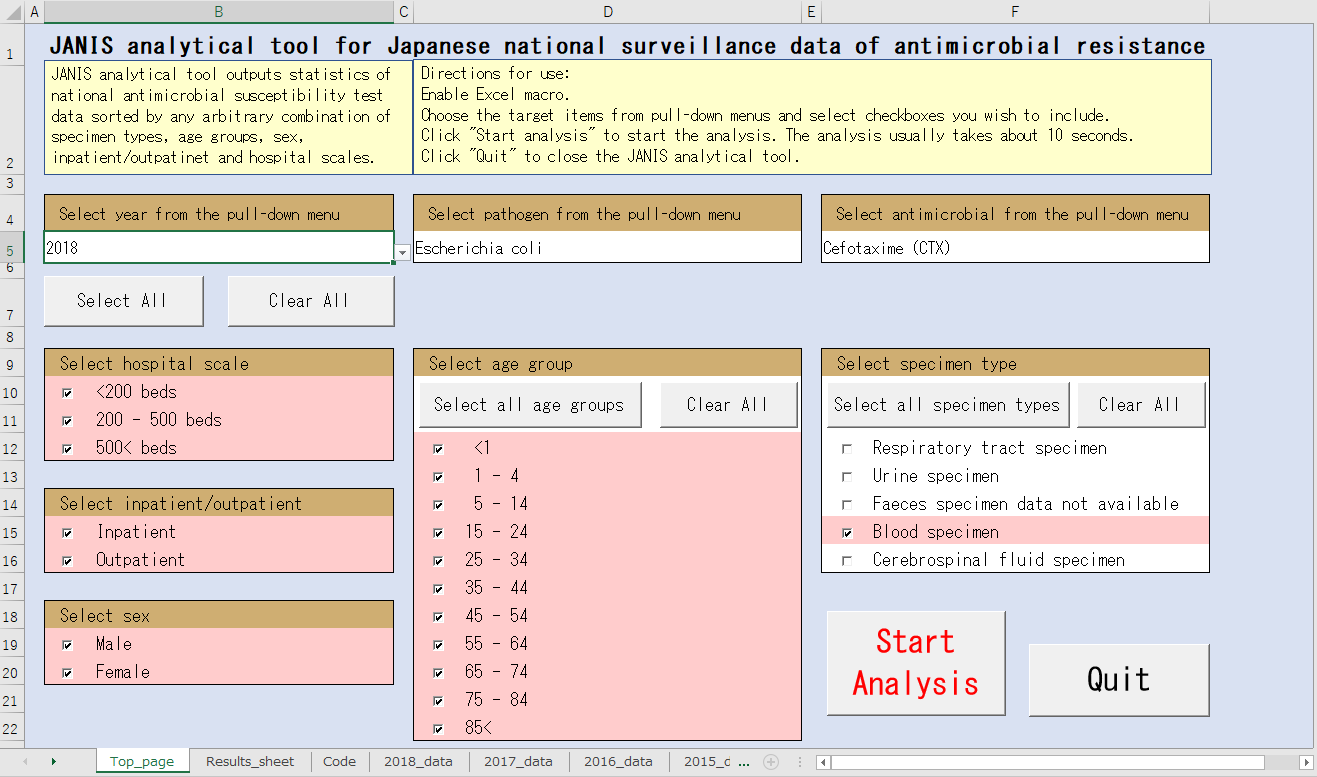


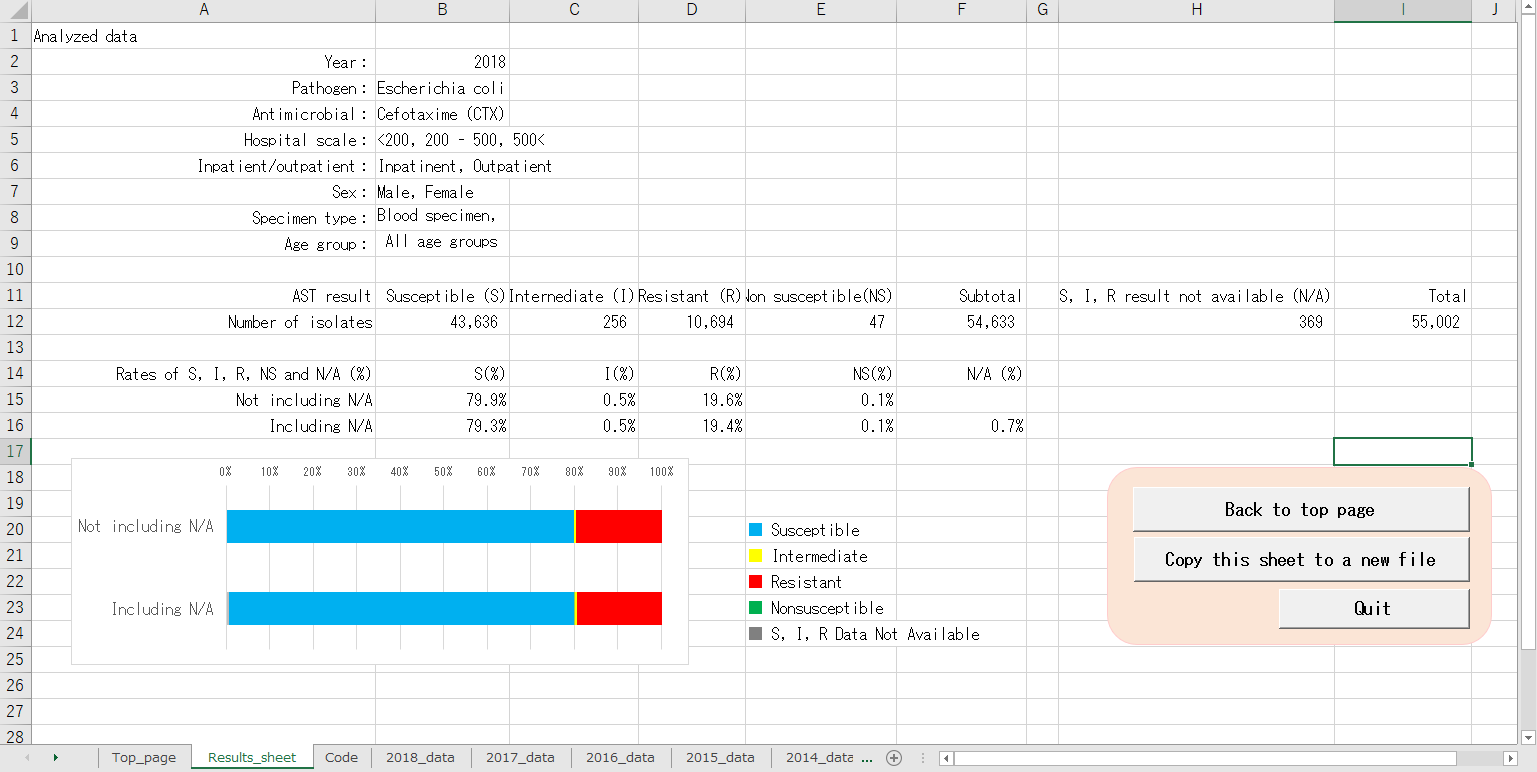


## Figure S1 – Graphical user interface and usage of the Excel tool.

It stores and can analyze the data tabulated using the de-duplication in GLASS. Users can interact with these data by simply clicking their mouse, and create antibiograms stratified by the bed size of hospitals (under 200, over 200 and under 500, over 500 beds), inpatient or outpatient, gender, specimen type, and age group as defined by GLASS. Based on this, users can create antibiograms after excluding UNKNOWN_NO_AST. As another variable regarding missing data, GLASS defines UNKNOWN_NO_BREAKPOINTS representing the number of isolates where AST was performed but no interpretation of the results is available for a specific antibiotic. In the Excel tool, we have renamed this as UNKNOWN_NO_SIR to imply that S-I-R interpretation was not possible. It can be included or excluded from the denominator for calculating resistance rates in the tool.
